# New insights into a poorly known parasite, *Dero lutzi* (Oligochaeta: Naididae), associated with tree frogs of the genus *Scinax*: morphological evaluation and genotypic data

**DOI:** 10.1590/S1984-29612024005

**Published:** 2024-01-08

**Authors:** Isabela Caroline Oliveira da Silva, Priscilla Soares, Lennon Malta, Fernando Paiva, Vanda Lúcia Ferreira, Carina Elisei de Oliveira, Luiz Eduardo Roland Tavares

**Affiliations:** 1 Laboratório de Ciências Ambientais e Sustentabilidade Agropecuária, Universidade Católica Dom Bosco – UCDB, Campo Grande, MS, Brasil; 2 Laboratório de Parasitologia Animal, Instituto de Biociências, Universidade Federal de Mato Grosso do Sul – UFMS, Campo Grande, MS, Brasil; 3 Laboratório de Pesquisa em Herpetologia, Instituto de Biociências, Universidade Federal de Mato Grosso do Sul – UFMS, Campo Grande, MS, Brasil

**Keywords:** Anura, genetic data, morphology, oligochaete, parasite, Anura, dados genéticos, morfologia, oligoqueta, parasito

## Abstract

The oligochaete *Dero lutzi* follows a life strategy that alternates between free-living periods in aquatic environments and endoparasitic phases. Most occurrences of *D. lutzi* in anurans are reported in species with arboreal habits, with studies limited to the recording of the oligochaete’s presence in the host. Our study recovered specimens of *D. lutzi* from the tree frogs *Scinax fuscovarius* and *Scinax. nasicus*. We performed a morphological assessment of the parasite using light microscopy, for the first time, scanning electron microscopy. Molecular characterization of *D. lutzi* was carried out using the mitochondrial gene 16S rRNA and the nuclear gene 28S rRNA. Additionally, a phylogenetic tree was constructed to assess the species´position in relation to other group members. In our results, we confirmed the phenotypic morphological characteristics of the endoparasitic phase of *D. lutzi*. We also presented its phylogenetic position with other oligochaetes in the group, demonstrating the proximity between the endoparasite *D. lutzi* and the free-living oligochaete *D. superterrenus*.

## Resumo

O oligoqueta *Dero lutzi* possui uma estratégia de vida que alterna entre períodos de vida livre em ambientes aquáticos e fases endoparasitárias. A maioria das ocorrências de *D. lutzi* em anfíbios anuros é relatada em espécies com hábitos arborícolas, com estudos que se restringem a registros da presença do oligoqueta no hospedeiro. Este estudo recuperou espécimes de *D. lutzi* das pererecas *Scinax fuscovarius* e *Scinax nasicus*. Foi realizada uma avaliação morfológica do parasito por microscopia de luz e, pela primeira vez, por microscopia eletrônica de varredura. A caracterização molecular de *D. lutzi* foi realizada com o gene mitocondrial 16S rRNA e o gene nuclear 28S rRNA. Além disso, uma árvore filogenética foi construída para avaliar a posição da espécie em relação a outros membros do grupo. Os resultados deste estudo confirmaram as características morfológicas fenotípicas da fase endoparasitária de *D. lutzi*. Também foi apresentada sua posição filogenética com outros oligoquetas do grupo, o que permitiu demonstrar a proximidade entre o endoparasito *D. lutzi* e o oligoqueta de vida livre *D. superterrenus*.

## Introduction

Some species of Oligochaetes from the family Naididae have interspecific commensal, phoretic and parasitic relationships (Harman, 1971; Pinder et al., 1998; Gorni & Alves, 2006). Parasitic associations have been documented in aquatic animals, such as amphibians, fish, gastropods (Learner et al., 1978; Morais et al., 2017; Ingram et al., 2020). Oligochaetes of the genus *Dero* are known to have two types of associations with amphibians: phoresy (in which amphibians play a role in dispersion) and endoparasitic (Lopez et al., 1999; Sinsch et al., 2019). According to Andrews et al. (2015), the oligochaete *Allodero hylae* in its endoparasitic stage, can lead to intense infections that result in physical damage through the dilation of the kidneys and ureters.

*Dero lutzi* Michaelsen, 1926 is an endoparasite of frogs in Neotropical regions, with its free-living phases in exclusively aquatic environments (Oda et al., 2015; Morais et al., 2017). During the free-living period, *Dero* has gills at the posterior end of the body, in addition to dorsal bristles. The gills are usually enclosed in a chamber called the gill fossa, and they are essential structures for life in the aquatic environment (Harman & Lawler, 1975). When oligochaete species enter the host, the gills disappear, as they are structures unsuitable for the way of life as an endoparasite (Harman & Lawler, 1975; Oda et al., 2015). During the parasitic period, some oligochaetes can reproduce asexually within the host, and absorb resources for their survival (Pinder et al., 1998; Andrews et al., 2015). In this way, they can be expelled by the frog during the time of urination (Andrews et al. 2015).

Existing records of *D. lutzi* in tree frogs are restricted to South American countries (Brazil and Venezuela), with Brazilian occurrences in hosts in the states of Ceará, Mato Grosso do Sul, Minas Gerais, Paraná, Rio de Janeiro, Santa Catarina and São Paulo (Rodrigues & Maldonado, 1982 cited by Morais et al., 2017; Prado et al., 2003; Oda et al., 2015; Morais et al., 2017; Cañizales, 2020; Oliveira et al., 2022; Silva et al., 2022; Vrcibradic et al., 2023). In this study, we recorded *D. lutzi* in two tree frog species of the family Hylidae, which are *Scinax fuscovarius* Lutz, 1925 and *Scinax nasicus* Cope, 1862. They were captured in different regions of the state of Mato Grosso do Sul, Brazil. These species are widely distributed in South American countries, commonly found in areas of pastures, swamps and lakes in the rainy and dry seasons (Souza et al., 2017; Uetanabaro et al., 2007). In this context, we present complementary information on the morphological description of *D. lutzi* in its parasitic phase, using light microscopy and, for the first time, scanning electron microscopy. We also performed a molecular characterization of the species, using the mitochondrial 16S rRNA gene and the nuclear 28S rRNA gene.

## Material and Methods

### Collecting sites

The captures were carried out in three distinct regions of the state of Mato Grosso do Sul, Brazil (Serra da Bodoquena, Pantanal Sul-matogrossense and the Parque Estadual das Nascentes do Rio Taquari). Serra da Bodoquena is located on the edge of the Pantanal, in the mountainous region. In this location, we used the area of ​​Fazenda Estância Mimosa in the municipality of Bonito as one of the collection points (-20.980278°, -56.508889°, *datum*: WGS84). The site is of great conservation interest due to the partially preserved forest cover composed of several phytophysiognomies that provide shelter for many species, as well as the regional geomorphological feature represented by its altitude (Boggiani, 1999; Uetanabaro et al., 2007). In the Pantanal region, the collections were made in the sub-region of Nhecolândia, with searches carried out in the area of ​​Fazenda Nhumirim, in the municipality of Corumbá (-18.593556°, -56.371208°, *datum*: WGS84). The area is characterized by the predominant vegetation of savannas, floodplains, areas with ponds and extensive areas of pastures (Mioto et al., 2012). The third collection region was Parque Estadual das Nascentes do Rio Taquari, which is an Environmental Conservation Unit located in the northern region of the state of Mato Grosso do Sul (-17.98333°, 53.16666°, *datum*: WGS84). The Cerrado preserved area has grassland, forested regions and swampy areas (Felfili et al., 2004).

In the Serra da Bodoquena region, tree frog collections were carried out between October and November 2015 and between April and May 2016. In the Pantanal, they were collected in December 2014, March 2015, April and November 2016 and November 2017. In the Parque Estadual das Nascentes do Rio Taquari, samples were collected in January 2018 and January 2020.

### Sample processing

After capture, the specimens were transported to the laboratory and euthanized with 5% lidocaine (Xylocaina®, Aspen Pharma Indústria Farmacêutica Ltda, Serra, State of Espirito Santo, Brazil), which was distributed over the abdomen and absorbed through the skin. The sex of each tree frog was recorded and the snout–vent length (SVL) was measured with a digital caliper (accuracy of 0.1 mm). The body surface of each individual, the coelomic cavities and all internal organs were inspected under a stereomicroscope. The necropsied tree frogs were fixed in 10% formaldehyde, preserved in 70% alcohol and later deposited in the Zoological Reference Collection of the Federal University of Mato Grosso do Sul (*Scinax fuscovarius*: ZUFMS-AMP 04110- 04184, ZUFMS-AMP 13653- 13672; *Scinax nasicus*: ZUFMS-AMP 12365- 12422). The oligochaetes found were removed and stored in 70% alcohol for later identification steps, and after being identified they were also deposited in the zoological collection (*Dero* *lutzi*: ZUFMS- ANN00001).

### Morphological evaluation

For morphological diagnosis, the oligochaetes were mounted on temporary slides with Amann's lactophenol for clarification and visualization of their external characteristics and internal organs. The specimens were measured and photographically documented using a DM5500B LEICATM light microscope (Leica Microsystems, Wetzlar, Germany) with a digital image capture system using the LAS 3.8™ Leica® system software (Leica Microsystems, Wetzlar, Germany). The samples were identified by light microscopy and subjected to scanning electron microscopy (SEM), previously dehydrated with ethanol in progressive concentrations. Then, the samples were dried to a critical point, mounted on carbon tape and observed in a Hitachi model TM-3000™ scanning electron microscope (Hitachi, Chiyoda, Tokyo, Japan). All measurements of the oligochaetes were in millimeters, and the following characteristics were considered: total body length and width, number and length of body segments, locations of the mouth and anus as well as the shape, distribution, number and length of ventral bristles.

### Molecular evaluation

For DNA extraction, the DNeasy Blood & Tissue Kit (QIAGEN, Hilden, Germany) was used following the manufacturer's procedures and instructions. Extracted DNA was quantified using a NanoDrop™ 2000 spectrophotometer (Thermo Fisher Scientific®, Waltham, Massachusetts, United States). Two genes were partially amplified: mitochondrial 16S rRNA and nuclear 28S rRNA. The following pairs of primers were used: 16S Ann16SF (5′-GCGGTATCCTGACCGTRCWAAGGTA-3′) and Ann16SR (5′-TCCTAAGCCAACATCGAGGTGCCAA-3′) (Sjölin et al., 2005), and 28S 28SCI (5′-ACCCGCTGAATTTAAGCAT-3′) and 28SC2 (5′ GAACTCTCTCTTCAAAGTTCTTTTC-3′) (Dayrat et al., 2001). The polymerase chain reaction (PCR) parameters performed followed Sinsch et al. (2019). PCR products purified by enzymatic treatment with ExoProStar^TM^ (Cytiva©, Global Life Sciences Solutions USA LLC, Marlborough, MA, 01752, United States of America) and then sequenced by ACTGene (Ludwig Biotec, state of Rio Grande do Sul, Brazil) using the same sets of primers used in the PCR reactions.

The sequences were aligned and analyzed using the software Geneious™ version 9.1.5 (Geneious, 2020) and deposited in GenBank with accession numbers 16S rRNA MW027347 and 28S rRNA MW027615. A preliminary BLAST was performed on the GenBank database (Benson et al., 2013) in order to verify the relationship of proximity with other organizations representing *Dero*. The sequences used for the construction of a phylogenetic tree to estimate the positions of each species were those with the greatest similarity resulting from the BLAST performed initially, based on the choice of sequences and the study of Sinsch et al. (2019) (Table 1). The tree was rooted with *Trieminentia corderoi* based on the phylogeny of the group proposed by Sinsch et al. (2019). Sequences isolated from the same organism or clones with 100% genetic similarity were not included.

**Table 1:** Number of Genbank accessions in the 28S and 16S regions of endoparasite oligochaete species of the Naididae family.

**Species of parasitic oligochaetes**	**28S**	**16S**
*Allonais gwaliorensis*	KY633416.1	KY633311.1
*Allonais inaequalis*	KY633417.1	DQ459952.1
*Allonais paraguayensis*	GQ355439.1	GQ355399.1
*Branchiodrilus hortensis*	KY633420.1	KY633312.1
*Branchiodrilus semperi*	KY633421.1	KY633315.1
*Dero borellii*	KY633428.1	KY633324.1
*Dero digitata*	KY633425.1	DQ459954.1
*Dero furcata*	KY633427.1	KY633325.1
*Dero rwandae*	MN555443.1	MN215405.1
*Dero* sp.	GQ355446.1	GQ355407.1
*Dero superterrenus*	KY633429.1	KY633326.1
*Dero vaga*	KY633426.1	DQ459953.1
*Nais barbata*	KY633430.1	JQ424993.1
*Nais communis*	GQ355448.1	JQ424986.1
*Nais elinguis*	KY633443.1	JQ424980.1
*Rhyacodrilus coccineus*	GU902025.1	DQ459931.1
*Rhyacodrilus falciformis*	KX618795.1	KX618764.1
*Trieminentia corderoi*	KY633463.1	GU002447.1

The primers used in the study were chosen in accordance with Sinsch et al. (2019). Phylogenetic analyzes were based on the sequences formed by the concatenated 16S rRNA and 28S rRNA regions. The sequences were aligned using M-Coffee (Notredame et al., 2000) and then evaluated by the Transitive Consistency Score (TCS) (Chang et al., 2014). To verify the reliability of the aligned positions based on the values of the scores, the ambiguous aligned positions were adjusted. A partition homogeneity test was performed with PAUP software (Swofford, 2003) to verify whether the reconstruction could be elaborated with an evolutionary model for both concatenated regions. Due to the test result (*p*= 0.07), the regions were analyzed in a partitioned way, and thus, the data were subjected to analysis using MrBayes, to make Bayesian inferences (Huelsenbeck & Ronquist, 2001).

Evolutionary models and fixed parameters were chosen and estimated using Akaike’s information criterion with the jModelTest (Guindon & Gascuel, 2003; Darriba et al., 2012). Nodal supports were determined using the Markov Monte Carlo chain (2 runs, 4 chains) for 4 × 10^6^ generations with sampling every 4 ×10^3^ generations, and the first 25% of the sampled trees were discarded.

## Results

In the Serra da Bodoquena region, 75 specimens of *S. fuscovarius* were collected (males = 38, females = 31, juveniles = 6); in the Pantanal, there were 58 specimens of *S. nasicus* (males = 32, females = 20, juveniles = 6) and in the Parque Estadual das Nascentes do rio Taquari, there were 24 specimens of *S. fuscovarius* (males = 19, females = 3, juveniles = 2). All oligochaete specimens found were present in the urinary tract organs of the hosts, such as the bladder, kidneys and urethra. The tree frogs captured in the Serra da Bodoquena region showed a parasite prevalence of 2.66%, with an average intensity of 1,5. Specimens from the Pantanal Sul-matogrossense showed a prevalence of 5.7% and a mean intensity of 1.67 ± 0.35. In individuals captured in the Parque Estadual das Nascentes do Rio Taquari, the prevalence was 4.16%, with an average intensity of 5 ± 1.38. The largest number of oligochaetes found was 117 individuals present in the kidneys of a female specimen of *S. fuscovarius* (SVL = 37.73 mm) from the Serra da Bodoquena region. This outlier value has been excluded from the calculation of the average intensity to avoid skewing the values with unrepresentative heights.

### Morphological analysis

**Figure 1:**
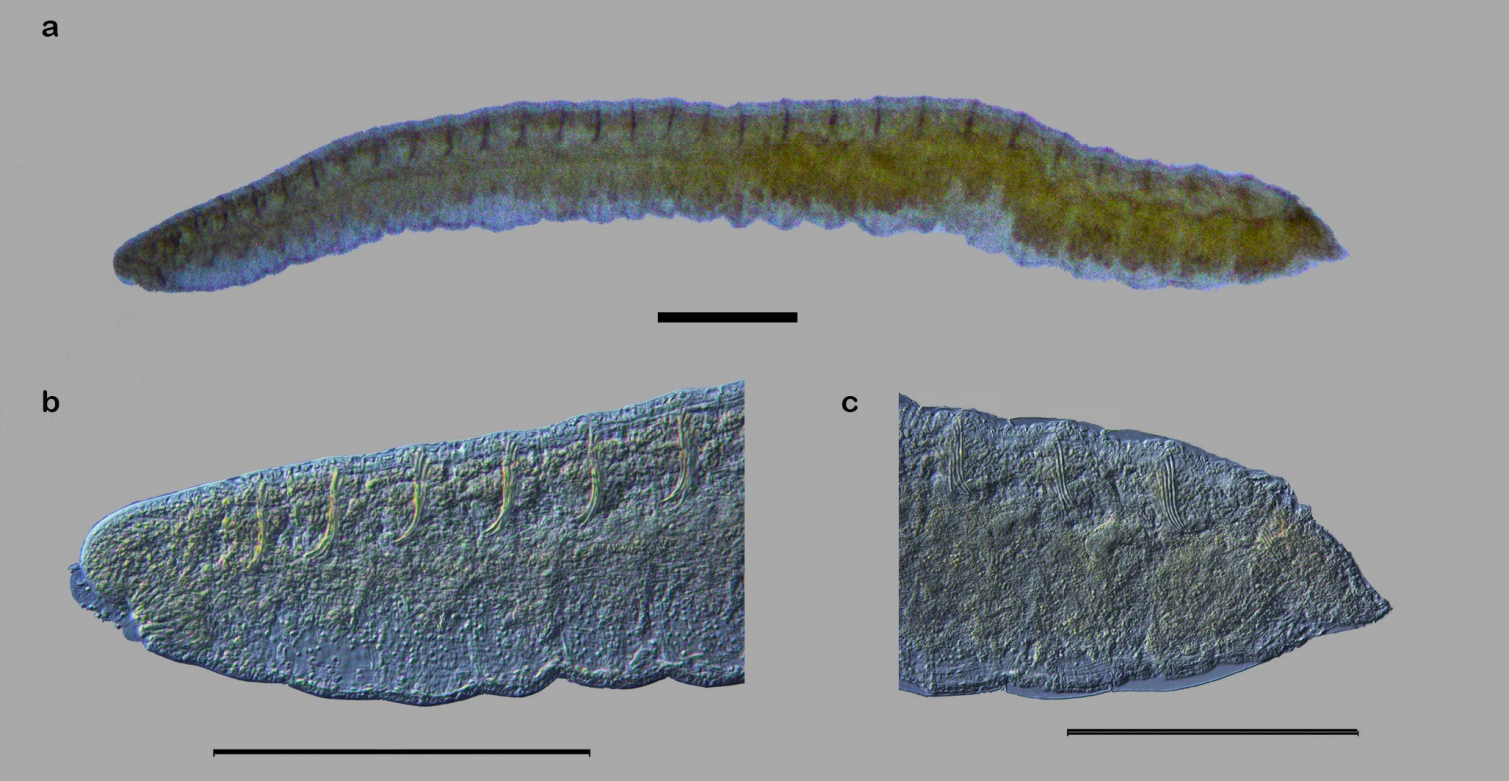
Light microscopy images of *Dero lutzi* in its endoparasitic stage. **a** Ventro-lateral view of the body of the endoparasite. **b** View of the posterior region. **c** View of the anterior region. All photos taken in Phase Interferential Contrast. Scale bar = 300 µm.

**Figure 2:**
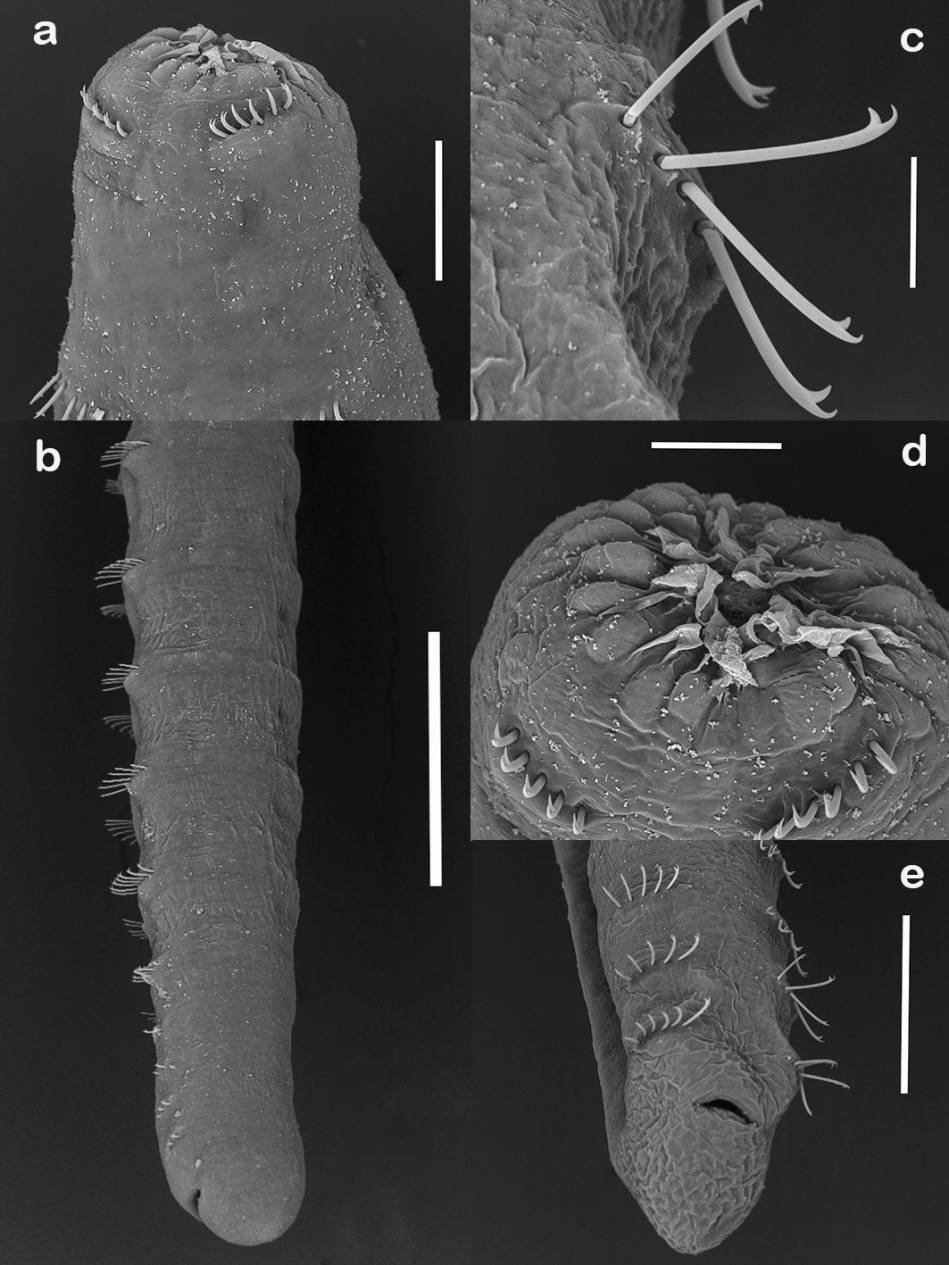
Scanning electron microscopy images of *Dero lutzi* in its endoparasitic stage. **a** Side view of the anterior portion (scale bar = 100 µm). **b** Posterior lateral view of the body (scale bar = 250 µm). **c** Detail of the pairs of setae distributed by the body segments (scale bar = 30 µm). **d** Oral opening and anterior region in apical view (scale bar = 25 µm). **e** Detail of the posterior region of the body (scale bar = 200 µm).

All oligochaetes found (n= 127) in the three collection areas were measured. The total body length of the specimens ranged from 3.85 to 5.49 mm, with a maximum width of 0.22 to 0.24 mm. The numbers of body segments ranged from 24 to 60, with widths from 0.24 to 0.26 mm. The mouth was located ventrally on body segment I; this segment does not contain ventral bristles, as they start from segment II. The bifid bristles had lengths of 0.08 to 0.09 mm and were organized into groups, with two groups in each body segment, in the amount of 4 to 6 bristles per group. These distributions were maintained until the posterior end of the body, where the anus is located (Figure 1). There were no dorsal bristles, gill fossae or even gills in the oligochaete when in a parasitic stage, which is a common characteristic among endoparasitic Nadidae (Harman & Lawler, 1975). There were also no eyes or internal organs in the endoparasite (Figure 2). The morphometric measurements and the characteristics observed in the examined specimens coincided with the diagnostic characteristics of previous studies for *D. lutzi* when it was found as an endoparasite in *S. fuscovarius* (Oda et al., 2015).

### Molecular analysis

**Figure 3:**
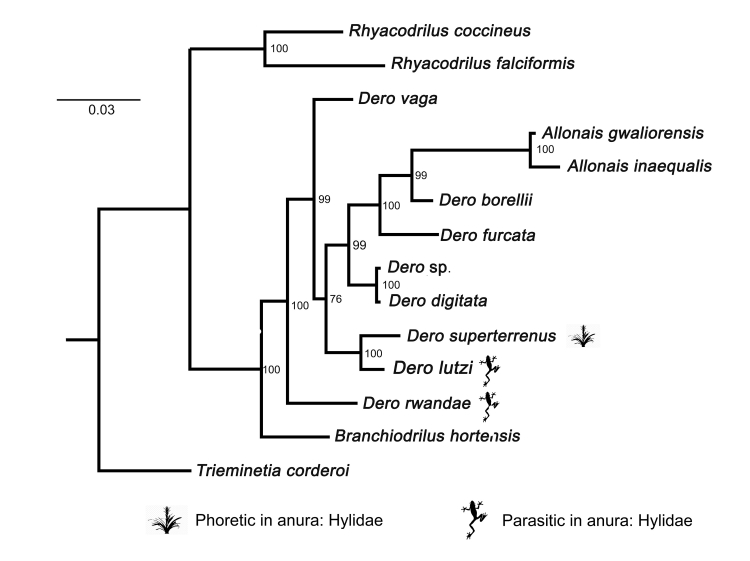
Phylogenetic tree of the regions 16S rRNA and 28S rRNA concatenated species of the family Naididae, generated with inference analysis Bayesiana.

The phylogenetic construction was performed based on Bayesian inference, with a total of 640 base pairs of the 16S rRNA (319 bp) and 28S rRNA (321 bp) regions concatenated. In this analysis, *D. lutzi* positioned itself as a sister group of *D. superterrenus* (Figure 3). *Dero* formed a non-monophyletic grouping with the genus *Allonais*, which is closely related to *Branchiodrilus*, *Dero* *borelli* and *Rhyacodrilus*, and formed two separate groupings of *Dero* and *Allonais* (Figure 3).

## Discussion

In this study, we documented additional information regarding the morphology and genotypic characterization of *D. lutzi* in its endoparasitic stage in anurans. The Serra da Bodoquena region exhibited a host with a high infection intensity in their kidneys, and the highest prevalence was found in the tree frogs collected in the Pantanal. In terms of morphological and morphometric characters, all observed measurements and features are consistent with previous records characterizing *D. lutzi* in its endoparasitic stage. Genotypic characterization analyses revealed the formation of distinct groups for *Dero* and *Allonais*, and also indicated that *D. lutzi* and *D. superterrenus* as sister groups.

When established as an endoparasite, the anatomical changes that allow infections to occur derive from the loss of characteristics used in the free-living environment, such as gills, dorsal bristles and gill fossae (Harman & Lawler, 1975). When the oligochaete comes into contact with its host, the parasite typically prefers to colonize the organs of the amphibian's urinary system, such as the urinary bladder, Wolffian duct, kidneys, and ureters (Pinder et al., 1998). During this stage, the oligochaete can feed on the host’s cells and multiply asexually by body fragmentation (Andrews et al., 2015). Massive post-infection multiplication can lead to high parasitic intensities in colonized organs, causing inflammation in the kidneys, ureters, and Wolffian duct (Andrews et al., 2015). This can result in the rupture of these organs due to the accumulation of parasites (Andrews et al. 2015). Although oligochaetes have found suitable habitats for their endoparasitic establishment in amphibians, their reproduction can be lethal to the host (Andrews et al., 2015).

In this study, an adult female *S. fuscovarius* collected in the Serra da Bodoquena region exhibited high intensity of *D. lutzi* in the kidneys, which may result from the seasonal multiplication of the parasites. According to Andrews et al. (2015), seasonal trends with peaks and decreases in abundance were observed for the endoparasite *Allodero hylae*. The age of the host is also a known characteristic that can lead to higher parasitic intensities. Adult individuals can provide larger spaces for parasite colonization, which contributes to a greater number of parasites in the host's organs (Ibrahim, 2008; Campião et al., 2015).

For the taxonomic classification of *Dero*, there are some controversies in the literature (Sinsch et al., 2019). *Dero* previously contained three subgenera (*Allodero*, *Aulophorus* and *Dero*); however in the study of Erséus et al. (2017), there was the inclusion of *Aulophorus* as a subgenus of *Dero*, and thus *Dero* was kept as a single genus. Recently Sinsch et al. (2019) described a new species of the putative subgenus *Allodero* in Rwanda in Africa by exploring the use of morphological and molecular data. The authors suggested that *Allodero* did not represent a separate evolutionary lineage from *Dero*, which invalidated this subgenus. Thus *Dero* does not have any valid subgenera. In our study, the results reinforced the invalidity of *Allodero*. They also corroborated the phylogenetic hypothesis of Erséus et al. (2017) and Sinsch et al. (2019), which showed *Dero* forming a non-monophyletic cluster with *Allonais*.

Another result obtained showed *D. lutzi* sharing its ancestry with the species *D. superterrenus* (Figure 3). *Dero superterrenus* Michaelsen, 1912 is a free-living oligochaete, with distribution in the Americas and Asia, and with records in Brazil in the states of Paraná, Pernambuco and São Paulo (Di Persia, 1980; Lopez et al., 2005; Christoffersen, 2007). The species is known to be widely found in bromeliad, and has been recorded in phoretic associations with amphibians inhabiting pond bromeliads and tree holes (Lopez et al., 1999, 2005). In this way, anurans can be used by the oligochaete so that it can disperse in some environments that are difficult to access, as several species of hylids use these places as shelter (Lopez et al., 1999; 2005; Moravec & Campos, 2020).

The free-living ancestor of *D. lutzi* and *D. superterrenus* may have displayed parasitic and/or phoric behaviors while exploiting the anurans. In addition, these behaviors are considered one of the routes that provide pathways for the establishment of parasitic associations (Roberts & Janovy, 2008). The constant exploitation of one organism by another, through its use as transport to disperse between habitats or even the use of this organism as a source of resources, can be considered factors that precede potential parasitic adaptations (Anderson, 2000; Morand et al., 2015).

## Conclusion

In this study, we confirmed the phenotypic morphological characteristics of the endoparasite stage of *D. lutzi*. We also present its phylogenetic position with the other oligochaetes of the group, a result that allowed the visualization of the existing proximity between *D. lutzi* and *D. superterrenus*.
